# Long polar fimbriae participates in the induction of neutrophils transepithelial migration across intestinal cells infected with enterohemorrhagic *E. coli* O157:H7

**DOI:** 10.3389/fcimb.2014.00185

**Published:** 2015-01-08

**Authors:** Alejandra F. Vergara, Roberto M. Vidal, Alfredo G. Torres, Mauricio J. Farfan

**Affiliations:** ^1^Departamento de Pediatría, Centro de Estudios Moleculares, Hospital Dr. Luis Calvo Mackenna, Universidad de ChileSantiago, Chile; ^2^Programa de Microbiología, Facultad de Medicina, Instituto de Ciencias Biomédicas, Universidad de ChileSantiago, Chile; ^3^Department of Microbiology and Immunology, Department of Pathology, University of Texas Medical BranchGalveston, TX, USA

**Keywords:** enterohemorrhagic *E. coli*, shiga toxin *E. coli*, long polar fimbriae, inflammation, PMN migration

## Abstract

Enterohemorrhagic *Escherichia coli* (EHEC) strains are causative agents of diarrhea and hemorrhagic colitis, both diseases associated with intestinal inflammation and cell damage. Several studies have correlated EHEC virulence factors to high levels of intestinal pro-inflammatory cytokines and we have previously described that the Long polar fimbriae (Lpf) is involved in the secretion of interleukin-8 (IL-8) and up-regulation of genes belonging to the NF-κB pathway using non-polarized epithelial intestinal T84 cells. In the current study, we evaluated the two EHEC O157 Lpf fimbriae (Lpf1 and Lpf2) for their ability to induce intestinal secretion of IL-8 and the activation of *IL8, CCL20*, and *ICAM1* genes on polarized T84 cells. We also determined the participation of Lpf1 and Lpf2 in transepithelial migration of polymorphonuclear neutrophils (PMNs). Polarized T84 cells infected with EHEC revealed that both, Lpf1 and Lpf2, were required for the secretion of IL-8 and the induction of *IL8, CCL20*, and *ICAM1* genes. Both fimbriae also played a role in the migration of PMNs trough the intestinal cells monolayer. Overall, the present work further demonstrated that the fimbriae Lpf1 and Lpf2 are important bacterial virulence factors that might be involved in the inflammatory responses associated with EHEC infections.

## Introduction

Enterohemorrhagic *Escherichia coli* (EHEC) is recognized as a food-borne human pathogen and an important etiological agent of bloody diarrhea in developing/industrialized countries and, in some instances, causative agent of hemolytic uremic syndrome (HUS) (Pennington, 2010). In humans, EHEC infection are characterized by the adherence of the bacteria to intestinal cells and the induction of an inflammatory process characterized by activation of the MAPK, AP-1, and NF-κB intracellular signaling cascades, leading to secretion of pro-inflammatory markers, including interleukin 8 (IL-8) (Dahan et al., 2002; Miyamoto et al., 2006).

Several virulence factors have been associated with the EHEC-mediated inflammatory process, including LPS, flagellin, and HCP pili (Farfan and Torres, 2012). Recently, we described that the Long polar fimbriae (Lpf) are one of the EHEC adherence factors mediating intestinal inflammation (Farfan et al., 2013). In EHEC O157:H7, two Lpf fimbriae has been described, Lpf1 and Lpf2 (Torres et al., 2002, 2004) and we found that the amount of IL-8 in the supernatant of cells infected by an isogenic EHEC *lpfA1 lpfA2* double mutant was significantly less as compared to cells infected with the wild type EHEC strain 86-24. Gene-expression analyses revealed that *IL1A* (Interleukin-1A), *TNF* (Tumor necrosis factor), *SELE* (E-selectin), *ICAM1* (Intercellular adhesion molecule-1), *IL8* (Interleukin 8), *CCL20* (Chemokine ligand 20), among other genes, were down-regulated in cells infected with the *lpfA1 lpfA2* double mutant (Farfan et al., 2013).

During the inflammatory response at the intestinal mucosa, neutrophils are key cellular components contributing directly in the resolution of the infection caused by enteric pathogens. The polymorphonuclear neutrophils (PMNs) migrate across the epithelial layer to reach the lumen and their recruitment is tightly controlled by chemokines and surface-exposed proteins produced by intestinal epithelial cells (Szabady and McCormick, 2013). IL-8, CCL20, and ICAM-1 are key proteins responsible for the chemoattraction and PMNs recruitment to the infection site (Coburn et al., 2005). Because the contribution of each Lpf fimbriae to the EHEC-induced inflammation of intestinal cells has not been evaluated, in this study we sought to evaluate the individual contribution of Lpf1 and Lpf2 in the induction of IL-8 secretion and also determine their involvement in the transepithelial migration of PMNs during EHEC infection using polarized intestinal cells.

## Materials and methods

### Bacterial strains and culture conditions

EHEC O157:H7 strain 86-24 and its isogenic mutant strains CDV468 (*lpfA1* mutant*)* (Torres et al., 2002), AGT201(*lpfA2* mutant) (Torres et al., 2004), and AGT210 (*lpfA1 lpfA2* double mutant) (Torres et al., 2004) were used in this study. All EHEC strains were grown overnight in Dulbecco's modified Eagle's medium (DMEM)/0.5% glucose (DMEM-HG) or Luria-Bertani (LB) broth with the addition of streptomycin (100 μg/ml), chloramphenicol (30 μg/ml) and tetracycline (12.5 μg/ml), when appropriate.

### T84 cell culture conditions

Human colonic T84 intestinal epithelial cells (ATCC CCL-248) were routinely maintained in DMEM-F12 media, supplemented with 10% fetal bovine serum (FBS), penicillin (10 U/ml) and streptomycin (10 μg/ml), at 37°C under 5% CO_2_.

### T84 polarized adherence assays

Adherence assay was performed with T84 cells in 12-well polycarbonate transwell filters with 0.4 μm pore (Corning) as described (Harrington et al., 2005). Medium was aspirated from the apical compartment and ~2 × 10^6^ bacteria were added to the monolayer. Plates were incubated at 37°C in 5% CO_2_ for 3 h and then washed 5× with PBS. Cells were lysed with a solution of 1% (vol/vol) Triton X-100 and the serial dilutions of the lysates were seeded on LB agar plates supplemented with streptomycin. The number of adherent bacteria was determined by enumerating the resulting colonies. For serum blocking assay, a solution containing ~2 × 10^6^ of bacteria strain 86-24 was incubated with serum raised against the Lpf1 or pre-bleed serum for 1 h at room temperature prior addition to the cell monolayer.

### Lpf fimbriae extraction

We obtained an enriched-fraction of fimbriae filaments as described (Izquierdo et al., 2014). Briefly, EHEC strains were grown in DMEM/HG at 37°C with shaking until an optical density at 600 nm (OD_600_) of 1.0 was reached. Cells were harvested by centrifugation at 6000 × g, re-suspended in 1.0 ml of a solution containing 0.5 mM Tris and 75 mM NaCl, and heated to 65°C for 30 min. Later, cells were pelleted by centrifugation at 6000 × g for 10 min and supernatants were recovered and centrifuged at 21,000 × g for 30 min to remove the remaining debris. Fimbriae extracts were dialyzed in PBS using Microcon® filter devices. Total protein was quantified and stored at −20°C for further analysis. To evaluate the induction of secretion of IL-8 of fimbrial extract in polarized T84 cells, each well was incubated with 200 μg of total protein.

### IL-8 secretion

Culture medium from triplicate wells of polarized T84 cell monolayers infected with EHEC obtained at 3 h after infection were evaluated in triplicate by ELISA for IL-8 as previously described (Harrington et al., 2005).

### Real-time PCR

Total RNA was obtained from polarized T84 cells infected for 3 h with EHEC strain 86-24 or its isogenic *lpf* mutants using Total RNA I (Omega Biotech) and treated with RNase-free DNase I (Omega Biotech). Two micrograms of RNA was reverse-transcribed into single-stranded by using kit AffinityScript™ qPCR cDNA (Stratagene). The synthesized cDNA was used to quantify the expression the *IL8, CCL20*, and *ICAM1* genes using the following primers: *IL8* F (ACT TCT CCA CAA CCC TCT GC), R (TCT GCA GCT CTG TGT GAA GG) (Sawa et al., 2008); *CCL20* F (TTT GCG CAC ACA GAC AAC TT), R (GCT GCT TTG ATG TCA GTG CT) (Gobert et al., 2007); *ICAM1* F (CCC ATT ATG ACT GCG GCT GCT), R (AGG CCA CCC CAG AGG ACA AC) (Sawa et al., 2008); *GAPDH* F (TCC ACC ACC CTG TTG CTG TA), R (ACC ACA GTC CAT GCC ATC AC) (Paulukat et al., 2001). The mRNA expression levels were normalized to those of the human housekeeping gene glyceraldehyde 3-phosphate dehydrogenase (*GAPDH*). Changes in cycle threshold (ΔCT) values for each gene were obtained by subtracting the mean threshold cycle (CT) of the reference *GAPDH* gene.

### Preparation of PMNs

Heparinized human blood was mixed with 1.5% dextran/0.9% NaCl and allowed to settle undisturbed for 30 min. The leukocyte-rich plasma was layered over a histopaque 1077 density gradient (Sigma) and centrifuged at 1300 × g for 30 min. The cell pellet containing PMNs and erythrocytes was treated with hypotonic lysis buffer and washed with HBSS buffer without Ca^2+^/Mg^2+^. The preparation contained ~95% neutrophils, as judged by morphological examination and cells were >90% viable, as determined by Trypan blue dye exclusion. PMNs were washed 3× and re-suspended in HBSS buffer without Ca^2+^/Mg^2+^ at 1 × 10^7^ cells/ml (Gibco). Prior to their use, calcein AM (1:1000, Becton Dickinson) was added to the suspension of PMN as described (Ruiz-Perez et al., 2011). The cells were incubated for 30 min at 37°C, and washed up to 3× with PBS before being re-suspended in 1 ml HBSS buffer without Ca^2+^/Mg^2+^.

### PMNs migration assay

Transmigration assays were performed according to previously described protocols (McCormick et al., 1993). Briefly, T84 cells were seeded on collagen-coated inverted inserts (diameter 12 mm; pore size 3 μm) at a concentration of ~7.5 × 10^5^ cells per insert in 100 μl of DMEM-F12 medium. The inserts were then placed in a 12-well plate and cultured until the monolayer were confluent (10–14 days). Prior to the infection, the cells were washed and incubated for 30 min a 37°C with DMEM-F12. The cells were infected on the apical surface with ~1 × 10^6^ of EHEC wild type strain 86-24 or its isogenic *lpf1* and *lpf2* single or *lpf1 lpf2* double mutants for 90 min at 37°C in 5% CO_2_. Neutrophil chemoattractant formyl-Met-Leu-Phe (fMLP) (0.2 μM, Sigma) was added as positive control. After infection, monolayers were washed 2 × in HBSS and 20 μl of solution containing ~1 × 10^7^ PMN/ml was added to the basolateral side. After 4 h, neutrophils that migrated into the lower chamber were collected for quantification with a fluorometer.

### Statistical analysis

Results from three independent experiments performed in triplicates were combined and analyzed by One-Way ANOVA with Dunnett posttest. A *P*-value of < 0.05 was considering statistically significant.

## Results

### Lpf1 and Lpf2 fimbriae participate in the induction of IL-8 secreted by polarized T84 cells

**Figure 1:**
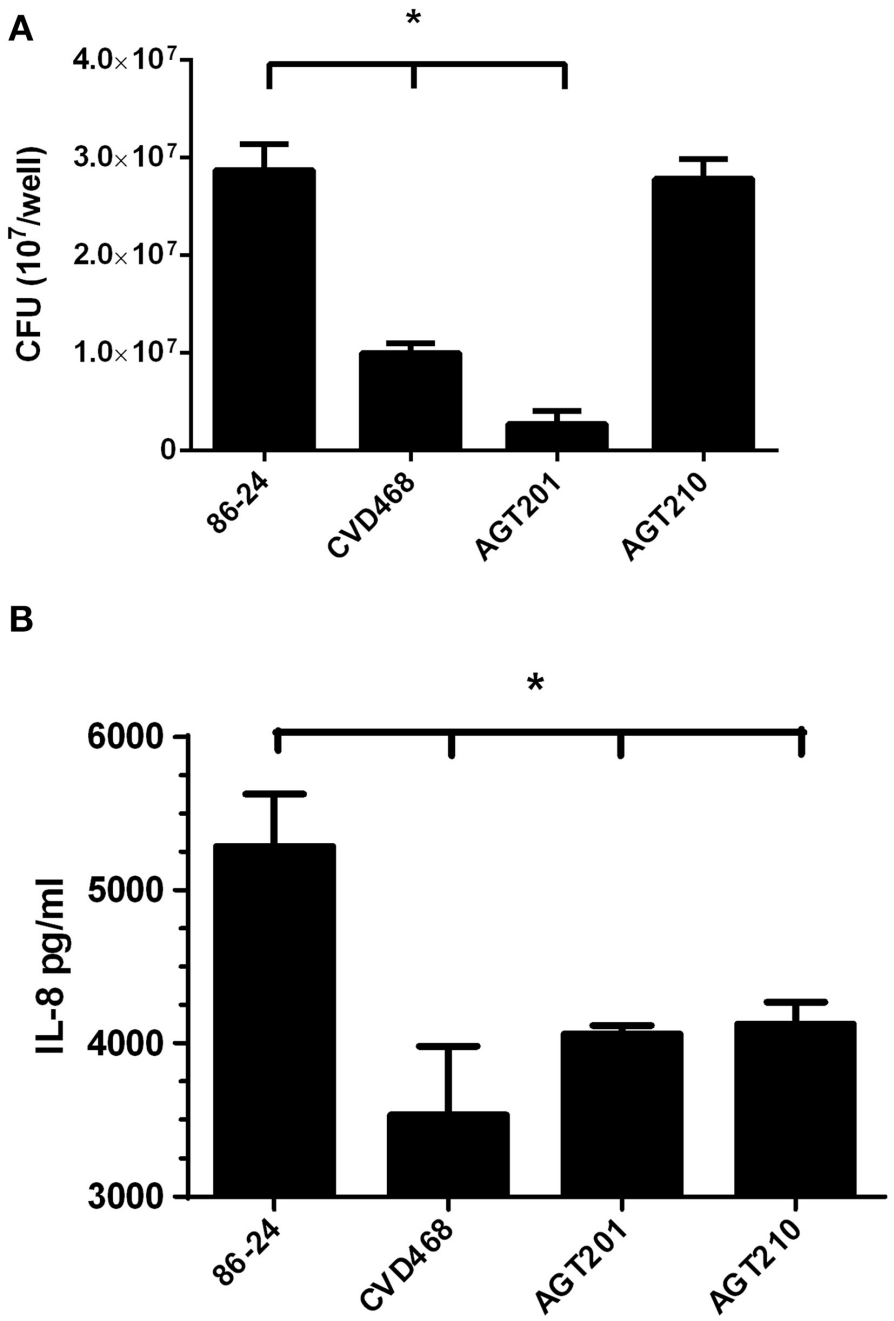
**Lpf fimbriae participate in the EHEC adherence and induction of IL-8 by polarized T84 cells**. **(A,B)** Polarized T84 cells were infected with the EHEC wild-type strain 86-24 and isogenic mutants in *lpfA1* (strain CVD468), *lpfA2* (strain AGT201), and *lpfA1 lpfA2* (strain AGT210). **(A)** After 3 h of infection the number of adherent bacteria was determined. **(B)** After 3 h of infection, infected cells were incubated with gentamicin for 3 h and the media from the basolateral side of the cells was collected and levels of secreted IL-8 were measured by ELISA. The bars represent the means of three experiments, with the error bars indicating one standard deviation. Asterisk represents statistical significance (*p* < 0.05).

We have previously demonstrated that Lpf fimbriae are involved in the adherence of EHEC to non-polarized intestinal epithelial T84 cells (Farfan et al., 2013). Using a polarized T84 cell model, a significant reduction in the number of adherent bacteria was observed on T84 cells infected with the *lpfA1* (strain CVD468) and *lpfA2* (strain AGT201) single mutants as compared to the wild-type strain. As previously observed with the non-polarized T84 cells, the number of adherent bacteria on polarized T84 cells infected by the *lpfA1 lpfA2* double mutant (strain AGT210) was comparable to that in cells infected with the wild-type strain (Figure 1A).

Next, the participation of the Lpf fimbriae in the secretion of IL-8 by polarized T84 cells was evaluated. Using cultured media from the basolateral compartment, it was found that the amount of IL-8 secreted by cells infected with the *lpfA1* and *lpfA2* single mutants, as well as *lpfA1 lpfA2* double mutant, was significantly lower compared to that in cells infected with the wild-type strain (Figure 1B).

**Figure 2:**
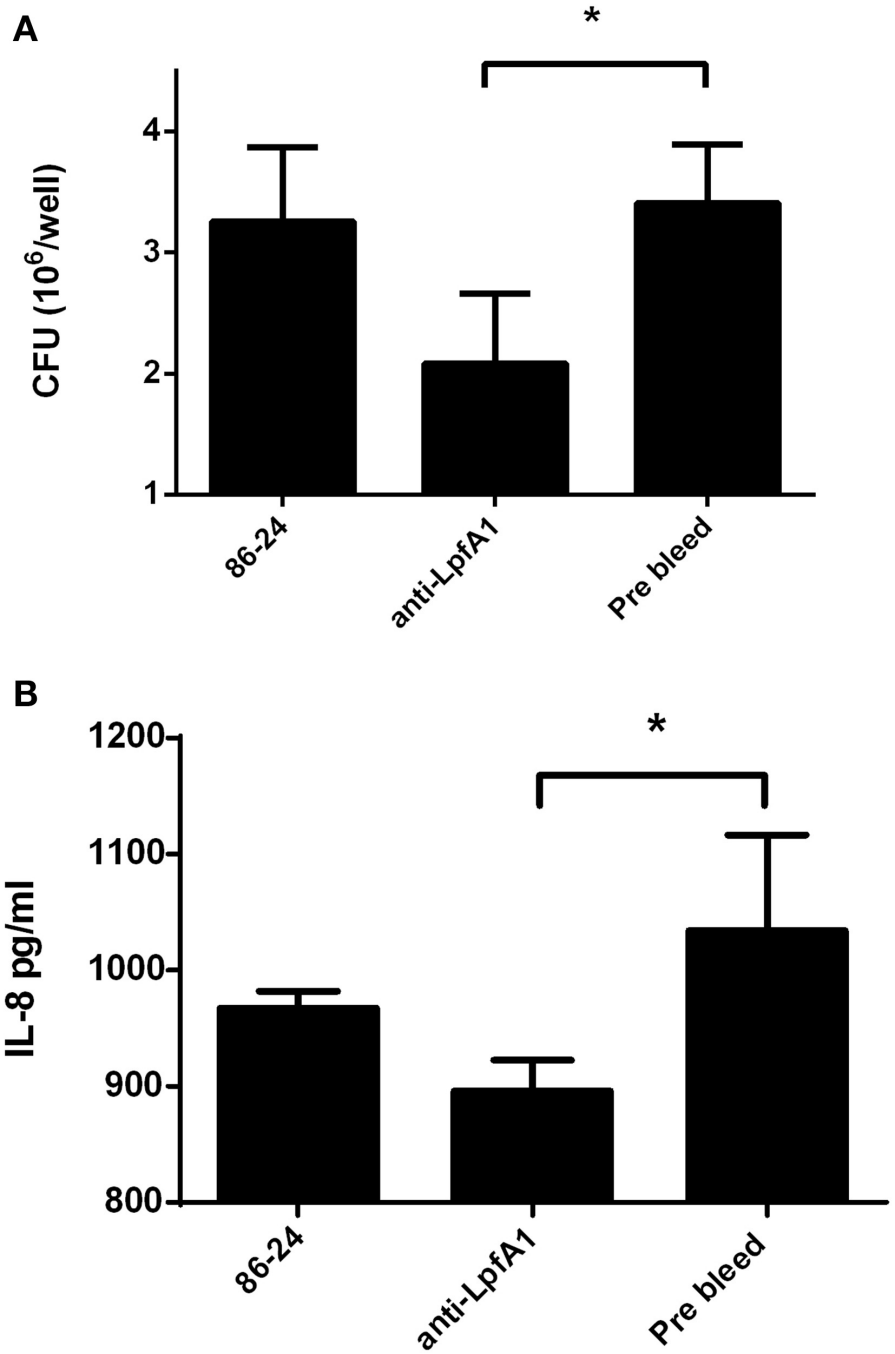
**Anti-LpfA1 serum blocked adherence to and secretion of IL-8 by T84 cells**. Polarized T84 cells were infected with the wild-type EHEC strain 86-24 incubated with anti-LpfA1 or pre-bleed serum. **(A)** After 3 h of infection the number of adherent bacteria was determined. The bars represent the means of three experiments, with the error bars indicating one standard deviation. ^*^Significantly different from adherence to T84 cells incubated with the pre-bleed serum and infected with the wild-type strain (*P* < 0.05). **(B)** After 3 h of infection, infected cells were incubated with gentamicin for 3 h and the media from the basolateral side of the cells was collected and levels of secreted IL-8 were measured by ELISA. Bars represent the means for three experiments, with error bars indicating one standard deviation. Asterisk represents statistical significance (*p* < 0.05).

Complementarily, we infected polarized T84 cells with wild-type EHEC strain in the presence of serum raised against the Lpf1 fimbrial major A subunit (LpfA1 protein) or pre-bleed serum as a control. A significant reduction in the number of adherent bacteria (Figure 2A) and secreted IL-8 (Figure 2B) was observed when EHEC wild type strain was incubated with anti-Lpf1 serum.

**Figure 3:**
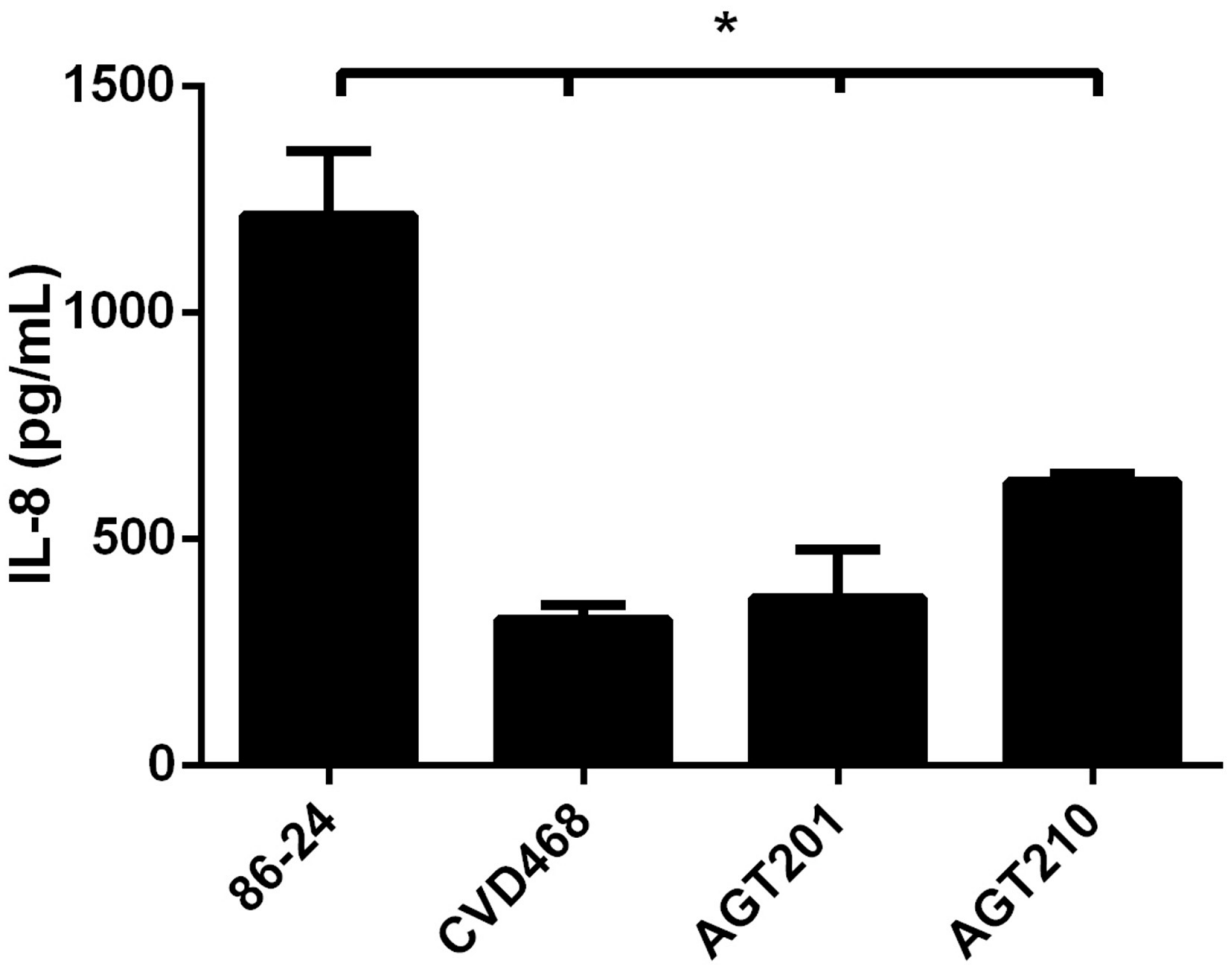
**Lpf fimbriae induce IL-8 secretion by T84 cells**. The T84 cells were incubated for 6 h with fimbrial extracts obtained the EHEC wild-type strain 86-24 and isogenic mutants in *lpfA1* (strain CVD468), *lpfA2* (strain AGT201), and *lpfA1 lpfA2* (strain AGT210). After the incubation, cells were incubated with media for 3 h and levels of secreted IL-8 were measured by ELISA. Bars represent the means for three experiments, with error bars indicating one standard deviation. Asterisk represents statistical significance (*p* < 0.05).

In addition, we incubated T84 cells with fimbrial extracts obtained from the wild-type EHEC strain 86-24 and its isogenic *lpf* mutants. The induction of IL-8 secretion in cells incubated with fimbrial extracts obtained from the mutant strains was significantly lower compared to those from the wild-type strain (Figure 3). Altogether, the data supported the participation of Lpf1 and Lpf2 fimbriae in the induction of the IL-8 secreted from intestinal epithelial cells.

### Lpf1 and Lpf2 participate in the up-regulation of *IL8, ICAM1* and *CCL20* genes

**Figure 4:**
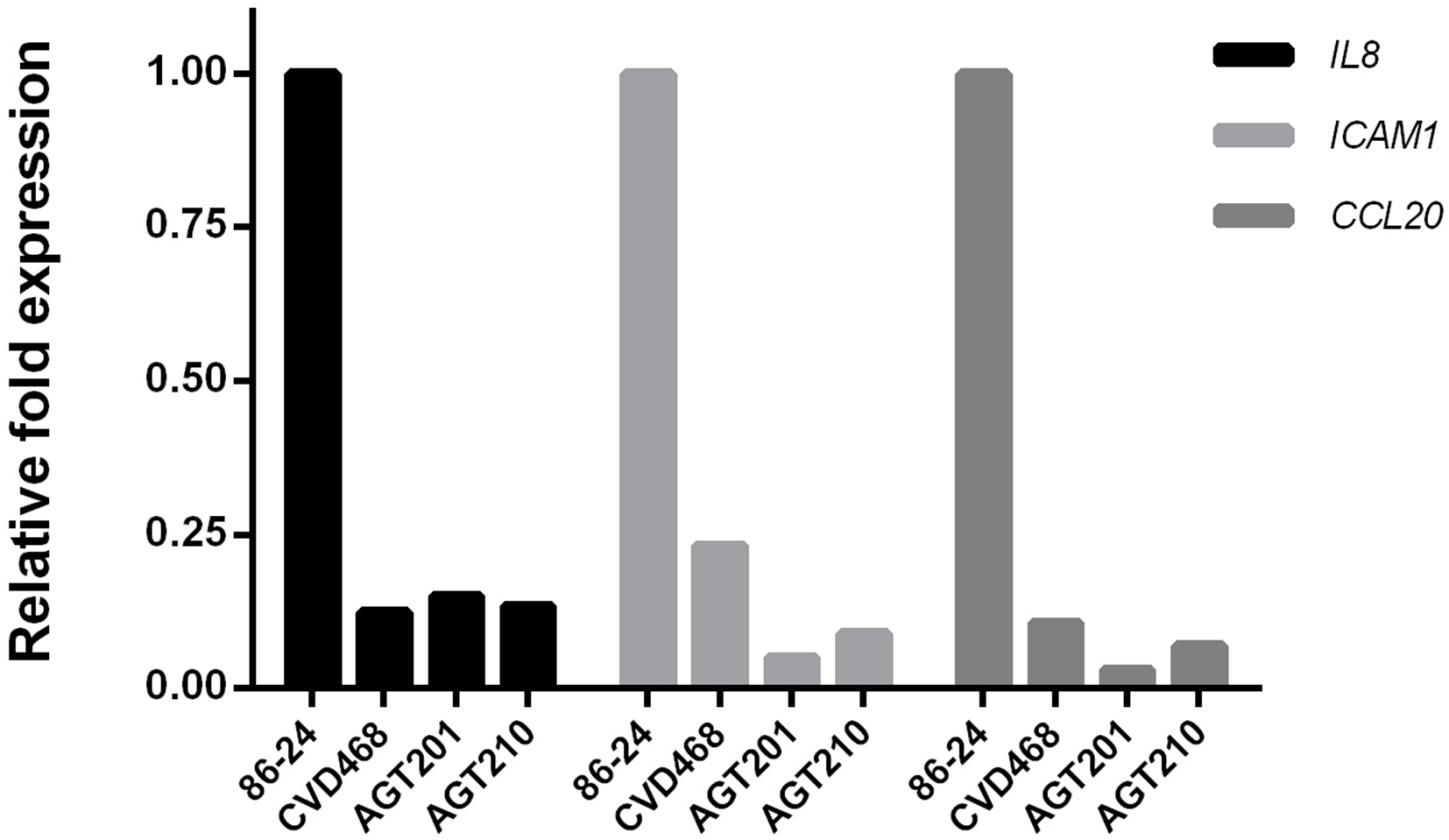
**Lpf fimbriae involvement in the expression of genes encoding inflammatory molecules**. Polarized T84 cells were infected with the EHEC wild-type strain 86-24 and isogenic mutants in *lpfA1* (strain CVD468), *lpfA2* (strain AGT201), and *lpfA1 lpfA2* (strain AGT210). After 3 h of infection, whole cell RNA was extracted to perform real-time RT-PCR analysis for *IL8, ICAM1*, and *CCL20* genes. The fold variation of gene expression was obtained by the comparative cycle threshold (Δ Δ C_T_) method, normalized with *GAPDH* constitutive expression gene.

It has been previously proposed that Lpf fimbriae are involved in the expression of several pro-inflammatory genes, including *IL-8, ICAM1*, and *CCL20* (Farfan et al., 2013). To determine the individual role of Lpf fimbriae in the expression of these genes, polarized T84 cells were infected with *lpfA1* and *lpfA2* single and double mutants. Real time PCR analyses indicated that Lpf1 and Lpf2 are equally involved in the expression of *IL-8* gene (~8 fold reduction compared to wild-type). In the case of *ICAM1* and *CCL20* genes, both fimbriae are also involved; however, a slight reduction in the expression of these genes was observed with polarized T84 cells infected with the *lpfA2* mutant compared those infected with the *lpfA1* mutant (Figure 4).

### Lpf fimbriae involvement in PMN migration across T84 monolayers

**Figure 5:**
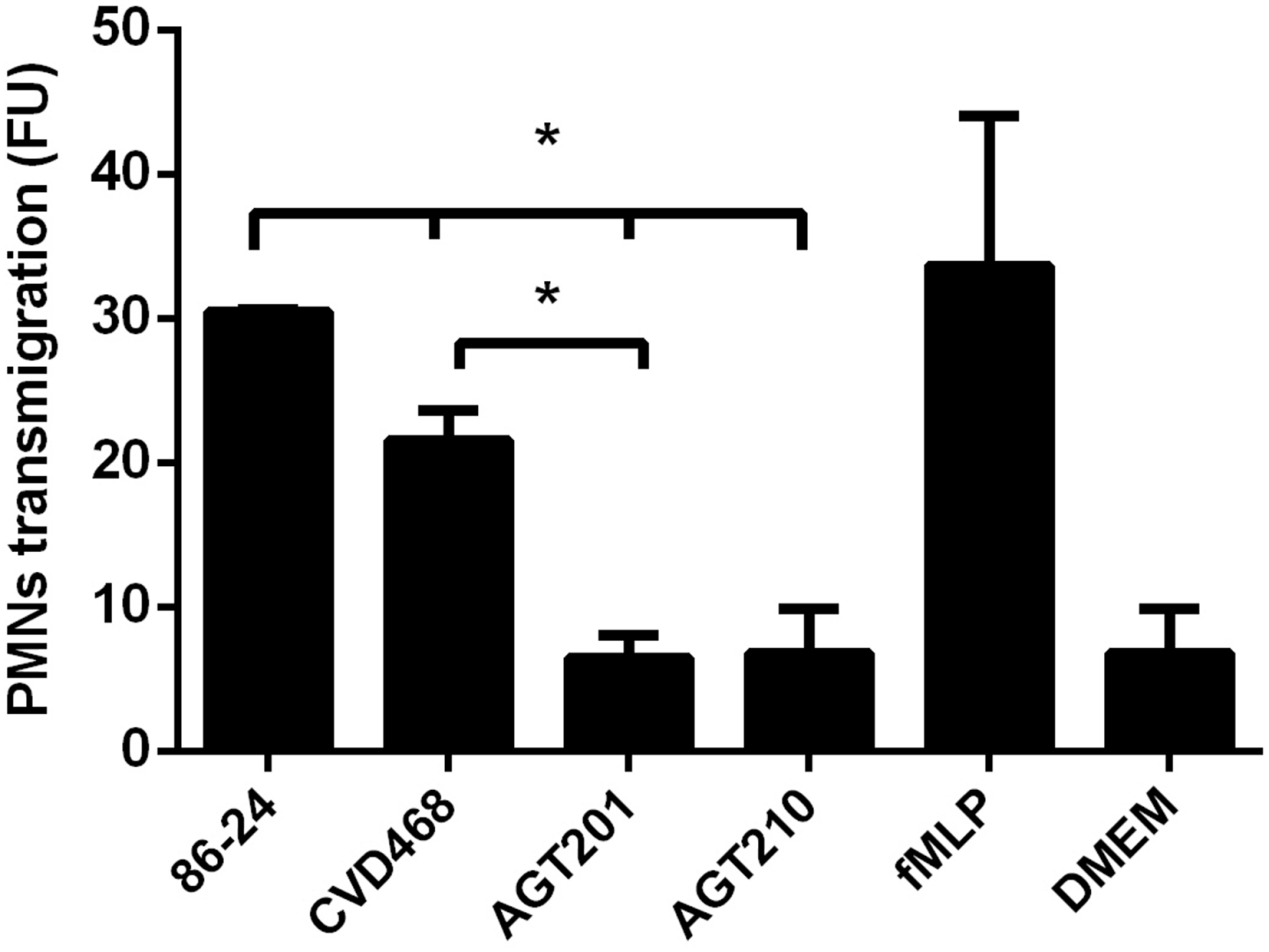
**Participation of Lpf fimbriae in the stimulation of PMN transepithelial migration**. Inverted polarized T84 cells were infected with the EHEC wild-type strain 86-24 and isogenic mutants in *lpfA1* (strain CVD468), *lpfA2* (strain AGT201), and *lpfA1 lpfA2* (strain AGT210). After infection, cells were incubated with 1 × 10^6^ of calcein-AM labeled PMNs which were inoculated on the basolateral surface and penetration of cells through the membrane was measured fluorometrically after 4 h. Bars represent the means for three experiments, with error bars indicating one standard deviation. Asterisk represents statistical significance (*p* < 0.05). FU, arbitrary fluorescent units.

To characterize the role that Lpf fimbriae is playing in PMN transmigration, inverted polarized T84 cells were apically infected with wild-type EHEC strain, *lpfA1* and *lpfA2* single and *lpfA1 lpfA2* double mutants. Subsequently, calcein-labeled PMNs were added to the basolateral surface and the PMNs that migrated to the apical compartment were detected. Compared to wild-type, a reduced migration of PMNs was observed in polarized T84 cells infected with the *lpfA1* and the *lpfA2* single as well as the *lpfA1 lpfA2* double mutants. However, a significant difference was observed in the PMNs migration on cells infected with *lpfA2* single and *lpfA1 lpfA2* double mutants as compared to the *lpfA1* single mutant (Figure 5).

## Discussion

The inflammatory response induced by EHEC on intestinal cells involves the participation of multiple bacterial structures that interact with receptors at the cell surface and subsequent activation of intracellular signaling cascades. The NF-κB pathway is probably the most important signaling cascade involved in the inflammatory process induced by EHEC, as well as with other enteropathogens (Berin et al., 2002). Several EHEC virulence factors have been implicated in the activation of the NF-κ B. The flagella was associated with the induction of high levels of IL-8 compared to Shiga toxin (Miyamoto et al., 2006). Shiga toxin stimulates the secretion of IL-8, promoting a massive recruitment of leukocytes as result of the increase expression of adhesive molecules, such as E-selectin and ICAM-1 (Morigi et al., 1995). The Hemorrhagic coli pili (HCP) have been also shown to participate in the secretion of IL-8 and TNF-α induced by EHEC infection of epithelial cells (Ledesma et al., 2010). Previously, we proposed that the Lpf fimbriae, a major adherence factor in EHEC, also participate in the inflammatory process (Farfan et al., 2013). Here, we provide supportive evidence associating the role of Lpf fimbriae in the EHEC-induced inflammation on intestinal epithelial cells.

In this study, we have shown that the two Lpf fimbriae described in EHEC O157:H7 contribute similarly to IL-8 secretion by polarized T84 cells, as described previously in a non-polarized T84 cell model (Farfan et al., 2013). Using *lpf1* and *lpf2* single and *lpf1 lpf2* double mutants, we found that the secretion of IL-8 by infected polarized T84 cells was lower as compared to those infected with the wild-type strain (Figure 1B), Interestingly, there was no difference in the IL-8 levels found in supernatant of cells infected with the individual and double mutant strains, suggesting that both fimbriae contributed equally to the induction of IL-8 secretion. One plausible explanation of this phenotype might be the reduction of the number of adherent mutant bacteria attached to the surface of intestinal cells; however, the *lpf1 lpf2* double mutant strain adheres at the same level observed with the wild type strain (Figure 1A), arguing against this possibility. A deeper analysis of the double mutant strain revealed that increase expression of curli at the surface of the bacteria is responsible for restoring the adherent phenotype to intestinal cells (Lloyd et al., 2012), but, in our model, not in the induction of IL-8 secreted. T84 cells infected with the *lpf1 lpf2 csgA* (*csgA* gene encodes the major constituent of curli) triple mutant revealed that the amount of secreted IL-8 is similar as compared to the *lpf1 lpf2* double mutant (data not shown).

Additionally, infection of polarized T84 cells with the EHEC wild-type strain in presence of antiserum against the major subunit of the Lpf1 fimbriae, LpfA1, showed a significant reduction in the adherence and the induction of IL-8 (Figures 2A,B). We performed similar experiments using an antiserum raised against LpfA2 major subunit of Lpf fimbria; however, no significant reduction in adherence of the EHEC strain 86-24 to intestinal cells was observed, probably due to either low specificity of this antiserum to recognize LpfA2 protein as determined by Western blot analysis (data not shown) or the recognition by the antibody of protein epitopes no associated with the adherence phenotype. To overcome this situation, we obtained fimbriae enriched-fractions from all the EHEC strains tested and when T84 cells were incubated with fimbrial fractions obtained from the mutant strains, less secreted IL-8 was observed when compared to fimbrial extracts from the wild type strain (Figure 3). Our data supported the idea that both Lpf fimbriae participate in the stimulation of IL-8 secretion from intestinal epithelial cells, however, we cannot ruled out the contribution of these fimbriae in the stimulation of IL-8 secretion dependent on the Lpf-mediated adhesion to cells.

Several signaling pathways regulate the expression of the inflammatory genes (Dahan et al., 2002). In a previous work, we described that several genes of the NF-κB pathway (such as *IL8, ICAM1*, and *CCL20* genes) were down-regulated in T84 cells infected with the double mutant as compared to those infected with wild-type EHEC strain. However, these tests were performed using a PCR array (Farfan et al., 2013) and now we confirmed these observations in our polarized T84 cell model by quantification of the expression of these genes individually by real time PCR assay. Comparing the induction observed in cells infected with the wild-type strain, we found that the expression of *IL8, ICAM1*, and *CCL20* genes were down-regulated in cells infected with all mutant strains tested (Figure 4). Surprisingly, the expression of *ICAM1* and *CCL20* genes in cells infected with the *lpf2* mutant was lower compared to cells infected with *lpf1* mutant.

The inflammation process on intestinal cells induced by enteric pathogens stimulates the activation and secretion of several markers that result in the recruitment of PMNs and their migration, getting access to the infection site and eventually eradicating the pathogen (McCormick et al., 1993). IL-8, ICAM-1, and CCL-20 play a pivotal role in the intestinal inflammation and the recruitment of PMNs during enteric infection. For EHEC, previous studies have found that the wild type EHEC strain 86-24 is able to induce the migration of PMNs, but paradoxically, this migration process increase the translocation of the Shiga toxin, favoring the access of the toxin to the bloodstream and distribution to target organs (Hurley et al., 2001). PMNs migration process is apparently not related to Shiga toxin, intimin, or other gene products encoded in the LEE PAI, suggesting the participation of other virulence factors in this process (Hurley et al., 2001).

Considering the role of Lpf on inflammation and that pathogenic *E. coli* adherence factors involved in inflammation, such as the aggregative adherence fimbriae (AAF) of enteroaggreggative *E. coli*, have also been implicated in the PMNs migration (Boll et al., 2012), we evaluate the role of both Lpf fimbriae in the PMNs migration trough polarized T84 cells. Our results showed that the translocation of PMNs was significantly lower in cells infected with *lpf1* and *lpf2* single and double mutant strains as compared to cells infected with the wild-type strain (Figure 5). We also found that infection with the single *lpf2* and *lpf1 lpf2* double mutant, generated less PMN migration compared to single *lpf1* mutant, suggesting that Lpf2 fimbriae might have a more prominent role in the PMNs migration induced by EHEC. One possible explanation might be attributable to the differential gene expression of *ICAM1* and *CCL20* genes on polarized T84 cells infected with the *lpf1* and *lpf2* single mutants (Figure 4). Another possibility is the differential regulation controlling expression of these fimbrial structures. For Lpf1, it is known that the histone-like nucleoid protein (H-NS) binds to the regulatory sequence of the *E. coli* O157:H7 *lpf1* operon and “silences” its transcription, while Ler inhibits the action of the H-NS leading to expression of the *lpf1* operon (Torres et al., 2007; Rojas-López et al., 2011). For the *lpf2* operon, it has been recently demonstrated that bile salts, iron and the Fur protein participate in its expression (Arenas-Herníndez et al., 2014). It would be interesting to evaluate in the future protein expression of both Lpf fimbriae using different environmental conditions applied in our polarized cell infection model.

In conclusion, this report provides new evidence supporting the role of Lpf fimbriae in the induction of host pro-inflammatory responses to EHEC infection. Along with their well-studied function as adherence factors, Lpf fimbriae are multifunctional virulence appendages that might be considered in the design of new strategies to control the infection by this pathogen.

### Conflict of interest statement

The authors declare that the research was conducted in the absence of any commercial or financial relationships that could be construed as a potential conflict of interest.
